# ANXA1 Contained in EVs Regulates Macrophage Polarization in Tumor Microenvironment and Promotes Pancreatic Cancer Progression and Metastasis

**DOI:** 10.3390/ijms222011018

**Published:** 2021-10-13

**Authors:** Nunzia Novizio, Raffaella Belvedere, Emanuela Pessolano, Silvana Morello, Alessandra Tosco, Pietro Campiglia, Amelia Filippelli, Antonello Petrella

**Affiliations:** 1 Department of Pharmacy, University of Salerno, Via Giovanni Paolo II 132, 84084 Fisciano, Italy; nnovizio@unisa.it (N.N.); rbelvedere@unisa.it (R.B.); smorello@unisa.it (S.M.); tosco@unisa.it (A.T.); pcampiglia@unisa.it (P.C.); 2 Department of Pharmacological Sciences, Università del Piemonte Orientale, 28100 Novara, Italy; emanuela.pessolano@uniupo.it; 3 Department of Medicine, Surgery and Dentistry, University of Salerno, Via S. Allende 43, 84081 Baronissi, Italy; afilippelli@unisa.it

**Keywords:** annexin A1, macrophage polarization, pancreatic cancer, extracellular vesicles, tumor microenvironment

## Abstract

The tumor microenvironment (TME) is a dynamic system where nontumor and cancer cells intercommunicate through soluble factors and extracellular vesicles (EVs). The TME in pancreatic cancer (PC) is critical for its aggressiveness and the annexin A1 (ANXA1) has been identified as one of the oncogenic elements. Previously, we demonstrated that the autocrine/paracrine activities of extracellular ANXA1 depend on its presence in EVs. Here, we show that the complex ANXA1/EVs modulates the macrophage polarization further contributing to cancer progression. The EVs isolated from wild type (WT) and ANXA1 knock-out MIA PaCa-2 cells have been administrated to THP-1 macrophages finding that ANXA1 is crucial for the acquisition of a protumor M2 phenotype. The M2 macrophages activate endothelial cells and fibroblasts to induce angiogenesis and matrix degradation, respectively. We have also found a significantly increased presence of M2 macrophage in mice tumor and liver metastasis sections previously obtained by orthotopic xenografts with WT cells. Taken together, our data interestingly suggest the relevance of ANXA1 as potential diagnostic/prognostic and/or therapeutic PC marker.

## 1. Introduction

Pancreatic cancer (PC) correlates to poor prognosis and high mortality due to late diagnosis, as well as early stages. Resectable PC patients have poor prognosis due to several factors such as chemoresistance by tumor microenvironment (TME) and by tumor cells per se [1]. Recent studies show that TME plays a critical role in PC progression [2,3], highlighting the strong relationship between the microenvironment and metastasis. The TME includes the extracellular matrix, cellular elements such as cancer cells, activated fibroblasts (carcinoma-associated fibroblasts, CAFs), endothelial and immune cells and extracellular components such as vesicles, which together play a predominant role in cancer development and chemoresistance [3,4].

In TME, macrophages, indicated in this case as tumor-associated macrophages (TAMs), represent the major immune component and are crucial for cancer progression. Most macrophages originate from circulating monocytes and form a heterogeneous population. A network of molecules, factors and post-transcriptional regulators participate in directing macrophage polarization, discernible in classically activated macrophages M1 and alternatively activated macrophages M2 [5]. M1 macrophages are characterized by a proinflammatory phenotype, develop in response to lipopolysaccharides (LPS) or interferon-γ (INFγ) and release interleukin (IL)1β, tumor necrosis factor-α (TNFα), IL-6 or IL-12 to induce immune responses [4]. Instead, the M2 macrophages present an anti-inflammatory phenotype with an elevated expression of the mannose (CD206) and scavenger receptor (CD163) as well as a secretion of transforming growth factor-β1 (TGFβ1) and IL-10 to promote the remodeling of extracellular matrix and angiogenesis [6]. Macrophages polarize in M2 group in response to IL4 and IL13, and, particularly as TAMs, they acquire an M2-like protumor phenotype. M2 effects are balanced by the M1 antitumor group [7]. A marked expression of M2-markers in tumor tissues is correlated with a worse diagnosis and prognosis of cancer patients [8,9].

Annexin A1 (ANXA1) (37-kDa protein) exhibits calcium-mediated phospholipid-binding properties and participates in many physiopathological processes. Particularly, in addition to its well-known anti-inflammatory effects, this protein favors cell migration, differentiation and death according to its localization and mainly in a tissue-specific manner [10,11,12,13,14]. In addition, in tumors, ANXA1 levels and activity differ depending on the distinct tissue, and it is positively involved in cancer progression [14]. This protein is overexpressed in PC [15,16,17] and leads to the acquisition of a mesenchymal phenotype of both tumor and stromal cells, acting as an intra- and extra-cellular element. In this regard, ANXA1 is known to be externalized through various mechanisms including the vesicular structures [16,17,18,19] such as exosomes [19,20,21,22,23,24]. These latter (40–100 nm diameter) are small membranous extracellular vesicles (EVs) released from nonmalignant and malignant cells in the extracellular space and body fluids [21,25,26,27,28,29,30]. EVs play a central role promoting cell-to-cell communication, cell proliferation, migration and invasion and are involved in organizing premetastatic niches as key modulators of the tumor microenvironment [24,31,32,33,34,35]. Moreover, previous studies have shown that vesicles regulate the interactions between tumor and immune cells, such as TAMs [32,33]. There is limited knowledge about the role of ANXA1-EVs in macrophage polarization in TAMs-M2, as in PC. Thus, this study aims to demonstrate that the ANXA1 secreted by EVs expedites macrophage recruitment and promotes M2 macrophage polarization during PC progression. This subpopulation could have a strong impact on the other’s cellular components of tumor environment influencing each other and contributing to the tumor development.

## 2. Results

### 2.1. The Influence of PC Cell-Derived EVs on the Macrophage Polarization

**Figure 1:**
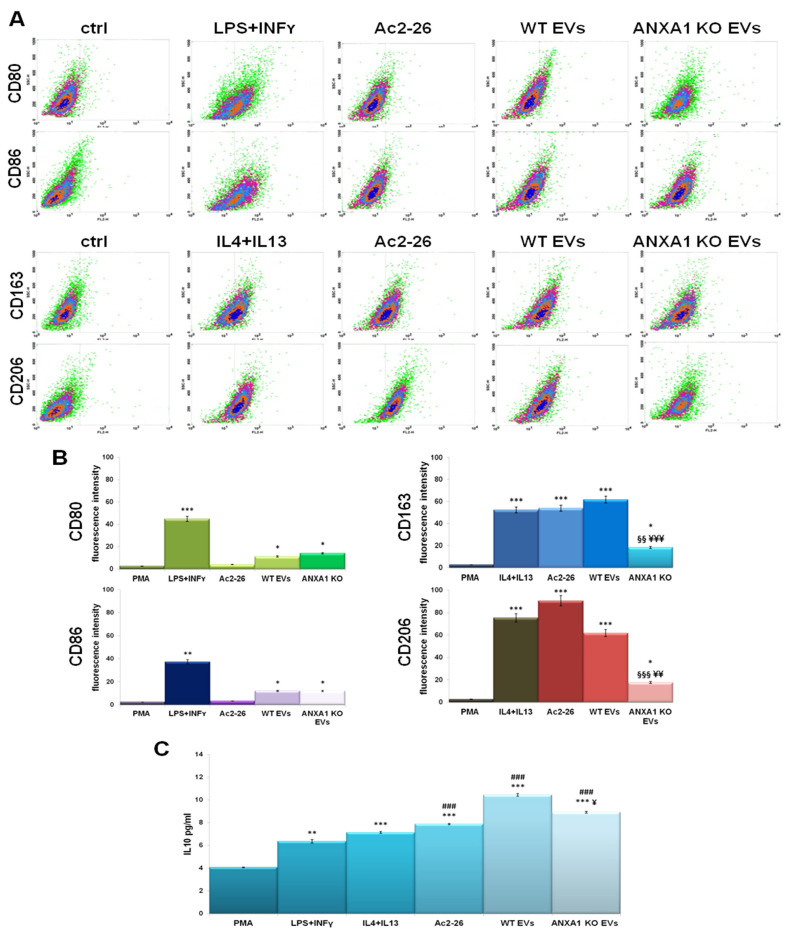
Macrophage polarization. (**A**) Flow cytometry analysis of M1 cell surface markers (CD80 and CD86) and M2 (CD163 and CD206) cell surface markers on THP-1 macrophages incubated with LPS+INF-γ (M1 induction cytokines), IL4+IL13 (M2 induction cytokines), Ac2-26 (1 µM) and WT and ANXA1 KO EVs for 24 h. (**B**) The histograms showed the expression of these markers on total cells analyzed through flow cytometry. (**C**) ELISA analysis of IL10 cytokine expression in THP-1 macrophages treated with LPS+INFγ (M1 induction cytokines), IL4+IL13 (M2 induction cytokines) and WT and ANXA1 KO EVs for 24 h. Data represent the mean of five independent experiments ± standard deviation with similar results. * *p* < 0.05; ** *p* < 0.01; *** *p* < 0.001 for treated cells vs. PMA treated controls; §§ *p* < 0.01; §§§ *p* < 0.001 for the point with ANXA1 KO EVs vs. Ac2-26; ¥ *p* < 0.05, ¥¥ *p* < 0.01, ¥¥¥ *p* < 0.001 for ANXA1 KO EVs vs WT EVs; # *p* < 0.05; ## *p* < 0.01; and ### *p* < 0.001 for each point vs. IL-4+IL-13.

In this work, we used the THP-1 cells, an immortalized cell line of human leukemic monocytes which maintains all the characteristics for studying monocyte/macrophage differentiation process since strongly showing M0/M1/M2 features. Thus, these cells have been pretreated with the phorbol-12-myristate-13-acetate (PMA), 320 nM for 6 h, which allowed the M0 polarization mainly confirmed by cell adhesion [35]. Then, the M0 population has been treated with a normalized amount of EVs derived from WT and ANXA1 KO MIA PaCa-2 cells [20]. After 24 h of treatment, THP-1 cells were harvested, and the surface markers were detected by flow cytometry to distinguish the two subpopulations: CD80/86 for M1 and CD163/206 for M2. Figure 1A displayed a strong polarization of macrophages treated with PC EVs into M1 or M2 subpopulation. In particular, WT EVs induced a high expression mainly of M2 markers compared to the treatment with IL-4 and IL-13 used as M2 controls. The administration of N-terminal mimetic peptide of ANXA1 (Ac2-26) proved that this differentiation is due to ANXA1. On the other hand, ANXA1 KO EVs were able to induce a phenotype switch in M1 population with high levels of its markers as seen in presence of LPS and INFγ, as technical M1 controls. The respective analyses of plots have been highlighted by histograms in Figure 1B. To confirm that the WT EVs induced the polarization of macrophages to M2 phenotype, we measured the production of the cytokine IL10 (a well-known M2 cytokine) by ELISA (Figure 1C). Actually, a significant increase in IL10 amount was found in PC cell-derived EVs-treated macrophages with respect of ANXA1 KO EVs and interestingly also more than the stimulus induced by the controls IL4 and IL13.

### 2.2. Effects of EVs from WT and ANXA1 KO MIA PaCa-2 Cells on Macrophage Migration and Invasion

**Figure 2:**
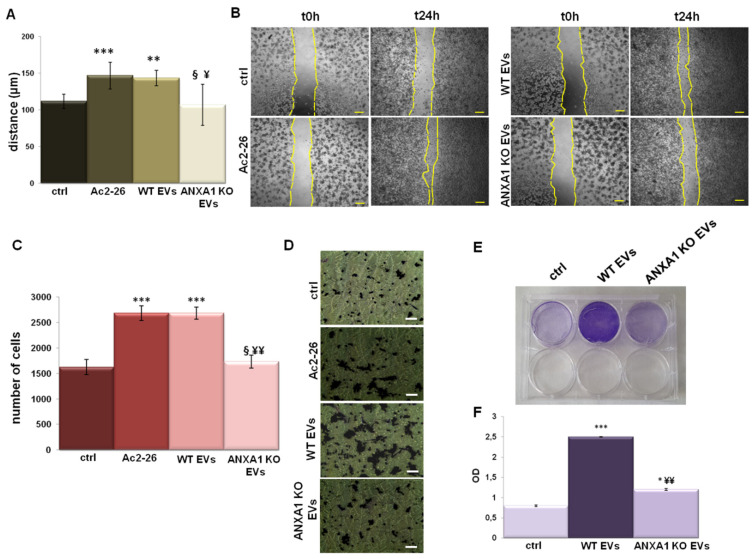
EVs effects on THP-1 macrophages. Analysis of (**A**) migration and (**C**) invasion speed of THP-1 macrophages treated with EVs from WT and ANXA1 KO MIA PaCa-2 cells with relative bright field images in (**B**,**D**), for migration and invasion, respectively. Bar = 50 μm. (**E**) Representative images of the clonogenic assay performed on THP1-macrophages in presence of WT and ANXA1 KO EVs for 24 h. (**F**) Histogram referring to the optical density (OD) obtained from 1% SDS cell dissolution and read to spectrophotometer. Data represent the mean of three independent experiments ± standard deviation with similar results. * *p* < 0.05; ** *p* < 0.01; and *** *p* < 0.001 for treated cells vs. PMA treated controls; § *p* < 0.05 for the point with ANXA1 KO EVs vs. Ac2-26; ¥ *p* < 0.05; and ¥¥ *p* < 0.01 for each point with ANXA1 KO EVs vs. WT EVs.

Here, we focused on the M0 macrophage motility assessing migration and invasion processes, particularly based on ANXA1 potential role. As shown in Figure 2A,C, cells migrated and invaded more rapidly in the presence of WT EVs than with ANXA1 KO EVs, both compared to PMA treated controls (in Figure 2B,D representative bright field images of wound healing and invasion assay, respectively, are reported). Ac2-26 confirmed that the exogenous ANXA1 was able to induce very similar effects of WT EVs. Furthermore, we have proved that the WT EVs promoted the proliferation of these M0 macrophages through a colony formation assay. We further distinctly reported as the ANXA1 KO EVs were less able to support cell growth. These results are described both in the bright field images (Figure 2E) and in the histogram analysis (Figure 2F) obtained from the SDS dissolution of cell-adsorbed crystal violet.

### 2.3. The Ability of ANXA1 to Affect the Tumor Microenvironment

**Figure 3:**
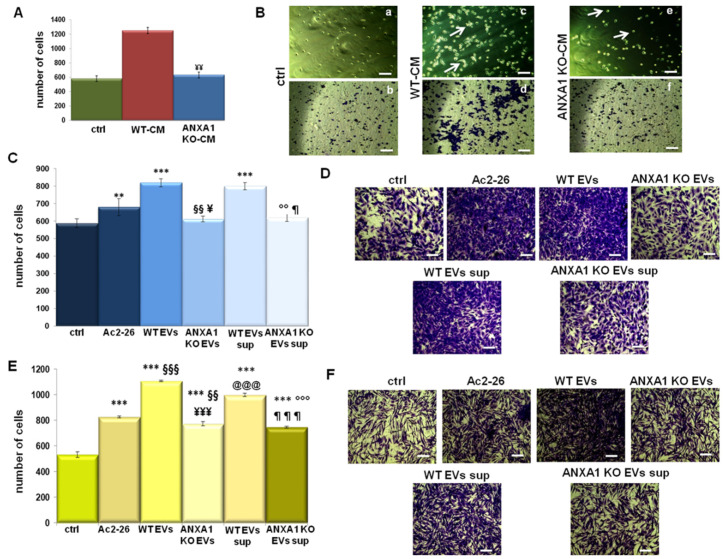
ANXA1 effects on TME. (**A**) Co-culture system performed by THP-1 macrophage invasion assay to WT and ANXA1 KO conditioned medium (CM). (**B**) Bright filed images of THP-1 cells captured in the bottom of the lower chamber of transwell (panels a, c and e) and representative images of macrophages in the same experimental points stained by crystal violet in the matrigel coating in the upper chamber of the generated invasion system (panels b, d and f). Results of (**C**) HUVEC and (**E**) BJ cells invasion to polarized M1 or M2 macrophages pretreated for 24 h with WT and ANXA1 KO MIA PaCa-2 EVs, with Ac2-26 (1 µM) and with the supernatants of THP-1 pretreated for 24 h with WT and ANXA1 KO MIA PaCa-2 EVs. The relative bright field images were shown in (**D**) for HUVEC and in (**F**) for BJ, respectively. Bar = 50 μm. Data represent the mean of four independent experiments ± standard deviation with similar results. ** *p* < 0.01; *** *p* < 0.001 for treated cells vs. PMA treated controls; §§ *p* < 0.01; §§§ *p* < 0.001 for each point with ANXA1 KO EVs vs. Ac2-26; ¥ *p* < 0.05; ¥¥ *p* < 0.01; and ¥¥¥ *p* < 0.001 for each point with ANXA1 KO EVs vs. WT EVs; °° *p* < 0.01; °°° *p* < 0.001 for ANXA1 KO EVs sup. vs. Ac2-26; ¶ *p* < 0.05; and ¶¶¶ *p* < 0.001 for ANXA1 KO EVs sup. vs. WT EVs sup; @@@ *p* < 0.001 for WT EVs sup. vs. Ac2-26.

In order to investigate the role of extracellular ANXA1, indirect co-culture systems were established between supernatants of WT and ANXA1 KO MIA PaCa-2 cells and macrophages and between EVs-treated macrophages and BJ or HUVEC, as described in Material and Methods section. Thus, we have demonstrated that the conditioned medium (CM) harvested from the WT MIA PaCa-2 cells induced the macrophage recruitment in a more significant manner if compared with the ANXA1 KO MIA PaCa-2 one. The histogram in Figure 3A refers to the panels a, c and e (Figure 3B), which represent the macrophages in the bottom of the lower chamber in response to CM from WT and ANXA1 KO cells. In the same Figure 3B, panels b, d and f are representative of the correspondent cells stained by crystal violet in the matrigel coating in the upper chamber of generated system. These last panels confirm that THP-1 cells invaded more rapidly in the presence of the WT cells CM compared to the ANXA1 KO cells one, which is in line with what we reported above. Furthermore, the white arrows indicate that in the presence of the CM from WT cells, the activated macrophages produced the typical ejections that are indispensible for the amoeboid movement capacity (Figure 3A, panel c). By contrast, when stimulated with ANXA1 KO cells-CM, macrophages remained mostly with a rounded monocyte-like appearance, such as the untreated condition (Figure 3A, panel e and a, respectively).

Finally, we have studied that polarized macrophages, obtained by the pretreatment with WT and ANXA1 KO EVs, were differently able to recruit fibroblasts and endothelial cells. To confirm the peculiar role of the extracellular ANXA1 in this process, we also used M0 macrophages treated with Ac2-26 as a positive control. Figure 3B,C shows the invasion of HUVEC and BJ, respectively. In both cases, as evidenced by histograms and bright field images, cell enrolment was significantly enhanced by M2 macrophages compared to M1 ones and mainly to the PMA-treated control. These results were also confirmed using the supernatant (sup.) of M1 and M2 macrophages polarized by WT and ANXA1 KO EVs. Therefore, we studied the ability of polarized macrophages in M2, following the pretreatment with WT EVs for 24 h, and M1, thanks to ANXA1 KO ones, to activate BJ and HUVEC motility.

### 2.4. The Influence of WT MIA PaCa-2 EVs on Endothelial Cell Activation

**Figure 4:**
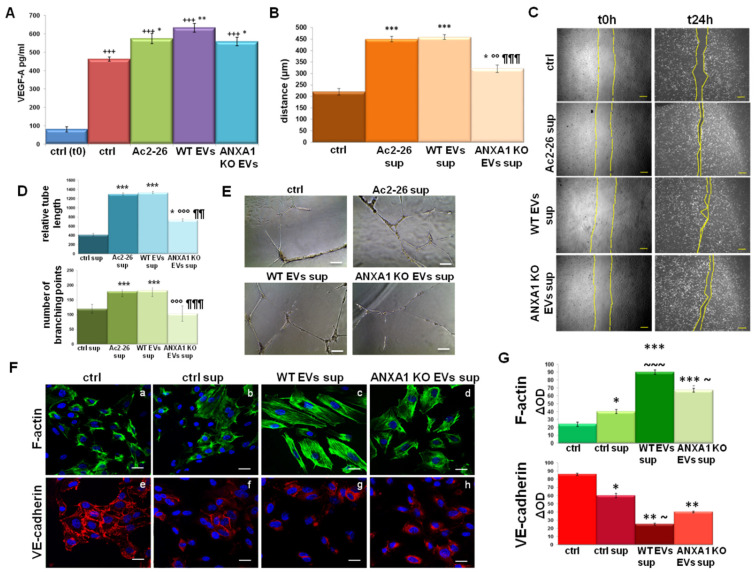
Endothelial cells activation induced by M2 macrophages. (**A**) ELISA analysis of VEGF-A expression in the supernatants of macrophages treated with Ac2-26 (1 µM) and WT and ANXA1 KO EVs for 24 h. Result of HUVEC (**B**) migration and (**D**) in vitro angiogenesis in presence of THP-1 supernatants pretreated for 24 h with WT and ANXA1 KO MIA PaCa-2 EVs and Ac2-26 (1 µM). The relative bright field images are reported in (**C**) for migration assay (Bar = 50 μm) and in (**E**) for angiogenesis one (Bar = 100 μm). (**F**) Confocal analysis for HUVEC cells: F-actin (panels a–d) and VE-cadherin (panels e–h) with the related densitometry evaluation in (**G**). Nuclei were stained with DAPI 1:1000 for 30 min at RT in the dark. Magnification 63 × 1.4 numerical aperture (NA). Bar = 100 μm. Data represent the mean of three independent experiments ± standard deviation with similar results. * *p* < 0.05; ** *p* < 0.01; and *** *p* < 0.001 for treated cells vs. untreated controls; ¥¥ *p* < 0.01; ¥¥¥ *p* < 0.001 for each point with ANXA1 KO EVs vs. WT EVs; +++ *p* < 0.001 for each experimental point vs. baseline control t0 meaning the treatment exclusively with PMA (320 nM) for 6 h; °° *p* < 0.01; °°° *p* < 0.001 for ANXA1 KO EVs sup. vs. Ac2-26 sup; ¶¶ *p* < 0.01; ¶¶¶ *p* < 0.001 for ANXA1 KO EVs sup. vs. WT EVs sup; ~ *p* < 0.05; ~~~ *p* < 0.001 for ANXA1 KO EVs sup. and WT EVs sup. vs. ctrl sup.

Next, we have investigated how polarized M1 or M2 macrophages could influence the activation of endothelial cells and promote the angiogenesis. Since the vascular endothelial growth factor-A (VEGF-A) has one of the major mediators of angiogenesis, we measured its production by ELISA finding a notable increase in the supernatant of macrophages treated with EVs. In particular, WT EVs acted more efficiently than ANXA1 KO ones with a significant difference, both when compared to each other and compared to control, both at baseline (only following treatment with PMA for 6 h) and after 24 h more from the seeding with growth medium. Interestingly, M2 macrophages with WT EVs have been shown to be able to secrete more VEGF-A than ANXA1 KO EVs treated. Additionally, the positive effect of Ac2-26 about the induction of VEGF-A section has been strongly evaluated (Figure 4A). Later, we focused on cell motility evaluating that HUVEC migrated more rapidly if treated with supernatant of M2 macrophages (WT sup—obtained from macrophages after a pretreatment with WT EVs) compared to M1 supernatant (ANXA1 KO sup—harvested from differentiated THP-1 pretreated with ANXA1 KO EVs) (Figure 4B,C for representative images). Furthermore, also for the in vitro angiogenesis the capability of macrophage supernatant to strongly stimulate this process was confirmed. Notably, the WT EVs sup. promoted a significant number of branching points and the relative tube length compared to ANXA1 KO EVs sup. and the untreated control (Figure 4D,E for bright field images). The macrophage supernatant containing Ac2-26 (Ac2-26 sup.) again confirmed its positive action in a very similar manner to WT EVs sup. stimulus (Figure 4D,E). Finally, based on the variation of HUVEC migration speed, we studied their cytoskeletal reorganization through confocal analysis. Thanks to phalloidin staining, we observed a well-organized cytoskeleton with more evident F-actin fibers in cells treated with WT EVs sup. when compared to other ones (Figure 4F, panels a–d). At this same experimental point, we inversely found that vascular endothelial (VE)-cadherin expression was significantly reduced compared to the control but also in respect to the ANXA1 KO EVs sup.-treated points (Figure 4F, panels e–h). In addition, in this case, we used as control the endothelial cells in the presence of their own growth medium (ctrl) and with HUVEC medium: macrophage growth medium 1:1 (ctrl sup). The experimental point with Ac2-26 was not explained because it was already shown in our previous study [34]. These results highlighted by the fluorescence images have been also proved by the histograms representing the densitometry analysis performed has reported in the Material and Methods section (Figure 4G).

### 2.5. The Support of TAMs on Fibroblast Activation

**Figure 5:**
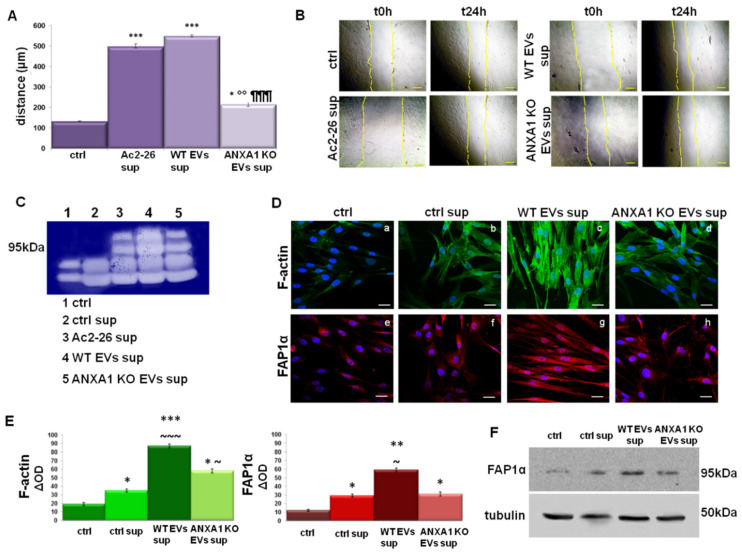
Fibroblasts activation induced by M2 macrophages. Results of (**A**) wound-healing assay and relative bright field images (**B**) of BJ cells with THP-1 supernatants obtained after 24 h with WT and ANXA1 KO MIA PaCa-2 EVs, and Ac2-26 (1 µM). Bar = 50 μm. (**C**) Gelatin zymography showing gelatinolytic activity of MMP-9 of BJ supernatants. (**D**) Immunofluorescence assay on BJ to detect: F-actin (panels a–d) and FAP1α (panels e–h) with the related densitometry analysis (**E**). Magnification 63 × 1.4 NA. Bar = 100 μm. (**F**) Western blot using antibodies against FAP1α on protein content of fibroblasts. Protein normalization was performed on tubulin levels. Data represent the mean of three independent experiments ± standard deviation with similar results. * *p* < 0.05; ** *p* < 0.01; and *** *p* < 0.001 for treated cells vs. untreated controls; °° *p* < 0.01 for ANXA1 KO EVs sup. vs. Ac2-26 sup; ¶¶¶ *p* < 0.001 for ANXA1 KO EVs sup. vs. WT EVs sup; ~ *p* < 0.05; ~~~ *p* < 0.001 for ANXA1 KO EVs sup. and WT EVs sup. vs. ctrl sup.

As shown in Figure 5A,B, the BJ cells migrated more rapidly in the presence of both Ac2-26 and of WT EVs sup. compared to those with ANXA1 KO EVs sup. and to untreated control. In order to support this result, we performed a gel zymography assessing the activity of metalloproteinases (MMPs) secreted by fibroblasts to degrade the extracellular matrix. Differently from MMP2 whose signal appeared overall unchanged, the MMP9 underwent a very prominent increase, after 24 h of WT-EVs sup. treatment as compared to the ANXA1 KO-EVs sup. (Figure 5C). Furthermore, activated fibroblasts are characterized by cytoskeletal changes, as revealed in this case through the marked increase in well-organized F-actin stress fibers and the acquisition of more precise and parallel directionally not only in respect to the control cells but also in respect to the ANXA1 KO EVs sup. treated cells (Figure 5D, panels a–d). Another important signal of fibroblast differentiation is the increase in the expression levels of specific protein markers—particularly relevant is the fibroblast activated protein 1α (FAP1α), a member of the group II integral serine proteases. BJ cells showed a notable increase in the levels of FAP1α expression when treated with WT EVs sup, more than ANXA1 KO EVs sup. (Figure 5D, panels e–h). As for HUVEC cells, we used two technical controls: the first one constituted by the BJ in presence of the growth medium and the second one by the BJ growth medium: macrophage growth medium 1:1 (ctrl sup). This information appeared evident in fluorescence images and through the densitometry analysis (Figure 5E). Moreover, the western blotting reported in Figure 5F, further confirms the different levels of FAP1α expression. 

### 2.6. Characterization of Macrophage Infiltration in WT and ANXA1 KO Tumor and Metastases

**Figure 6:**
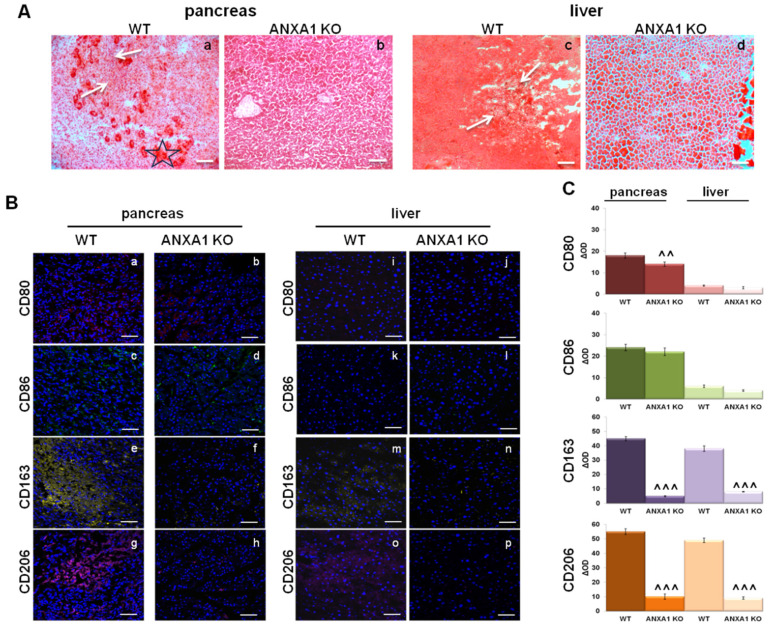
Macrophage infiltration in SCID mice sections derived from primary pancreatic tumors and livers after intrapancreatic implantation of WT and ANXA1 KO MIA PaCa-2 cells. In detail, we indicated as WT and ANXA1 KO pancreas the sections derived from mice in whose pancreas we have previously implanted WT and ANXA1 KO MIA PaCa-2 cells, respectively, and in which primary tumors have been developed. Additionally, WT and ANXA1 KO livers represented the hepatic sections in which we had found significantly more metastatic lesions deriving from tumors originating from WT MIA PaCa-2 cells compared to ANXA1 KO ones. (**A**) Pancreas (panels a and b) and liver (panels c and d) sections have been stained by H&E. Infiltrating ductal-like structures were labeled by star; macrophage infiltrations were marked by white arrows. Bar = 100 μm. (**B**) Representative images of CD80 (panels a and b; i and j), CD86 (panels c and d; k and l) (M1 macrophage markers) and CD163 (panels e and f; m and n) and CD206 (panels g and h; o and p) (M2 macrophage markers) staining by immunofluorescence in WT and ANXA1 KO mice pancreas and liver (livers in which we have found metastatic lesions derived from intrapancreatic injection of WT MIA PaCa-2 cells) sections. (**C**) The histograms showed the densitometry analysis on immunofluorescence images based on the intensity of each signal compared to the total number of nuclei. Nuclei were stained with DAPI 1:1000 for 30 min at room temperature (RT) in the dark. Magnification 40 × 1.4 NA. Bar = 100 μm. Data represent the mean of three independent experiments ± standard deviation with similar results. ^^ *p* < 0.01; ^^^ *p* < 0.001 for ANXA1 KO vs. WT cells xenografts.

TAMs are predominantly anti-inflammatory M2-macrophages. Thus, in order to assess the main phenotype, we analyzed through H&E staining the pancreas and liver tissue sections derived from mice in which we had previously performed intrapancreatic injections of WT and ANXA1 KO MIA PaCa-2 cells [15]. As reported, mice had been scarified after 5 weeks from the implantation, corresponding to the time when the primary tumor had become noticeably palpable for a week already. First, pancreas WT sections generally displayed multiple injuries, infiltrating ductal-like structures and extensive desmoplastic stromal reactions (Figure 6A, panel a, star), attributable to high macrophage infiltration (Figure 6A, panel a, white arrows) compared to the ANXA1 KO ones (Figure 6A, panel b). We also determined whether ANXA1 depletion could affect the stromal infiltration in metastasis formation. Therefore, we analyzed the liver section because it represents one of the first affected organs by the PC metastatic process. The H&E staining displayed a high infiltration of macrophages in WT livers (Figure 6A, panel c, white arrows), which were particularly notable in the metastatic niche in the section, signed by the lost structural integrity and reduced compactness. On the other hand, the ANXA1 KO liver sections retained their red color, integrity and tissue density and showed much less macrophage infiltration in metastasis lesion (Figure 6A, panel d). These results are in line with what we have previously found through macroscopic evaluation [15]. Next, in order to confirm the phenotype of TAMs in tumor and metastasis tissues, their characterization has been performed by an immunofluorescence assay. As shown in Figure 6B, a large number of CD206 and CD163 positive macrophages, more than CD80 and CD86, were observed in the WT pancreatic tumor and in the corresponding liver metastasis sections, suggesting the relevant presence of M2 macrophages. By contrast, low levels of CD206 and CD163 positive macrophages were detected in the ANXA1 KO tumor (panels e–h) and metastasis (panels m–p) tissues, where the main macrophage phenotype was the M1 one, as revealed by CD80 and CD86 signals (panels a, b and i, j for CD80; b, f and k, l for CD86; e, f and m, n for CD163; g, h and o, p for CD206). These results have been also confirmed by densitometry analysis shown in Figure 6C.

## 3. Discussion

Cancer-derived EVs, enriched in exosomes, are considered to be key components promoting cancer and microenvironment communication and play an important role in the modulation of the immune response [21,35,36,37,38,39,40,41,42]. It is known that EVs are able to influence stromal cells such as endothelial ones, fibroblasts, immune cells as lymphocytes and macrophages. In the present paper, we have investigated the effects of ANXA1, as cargo of EVs secreted by PC cells, on THP-1 highlighting its extracellular paracrine role in regulating the macrophages polarization in M2 subpopulation rather than in M1. These activities have been evaluated in terms of acquisition of a higher macrophages speed of migration, invasion and proliferation, all processes strongly involved in the formation of a TME particularly favorable for tumor progression [5,6,7,8,9,33,43,44,45]. The strong protumor role of ANXA1 has been proved by using its N-terminal peptide Ac2-26, able to mimic the main biological functions of the protein [19,46,47]. Moreover, this peptide also allowed us to show the secreted ANXA1 effects independently of the mechanism by which ANXA1 is externalized, since it is not yet defined if the protein is secreted only through vesicles. Furthermore, the EVs harvested from the ANXA1 KO MIA PaCa-2 have interestingly shown significant less impact about this action. About the extracellular activity of ANXA1, we have previously demonstrated that the complex ANXA1/EVs is able to activate endothelial cells and fibroblasts, as recipient stromal cells, inducing the acquisition of a more aggressive mesenchymal phenotype [20,35,48,49,50]. Furthermore, in PC progression, ANXA1 has been already associated to a strong oncogenic action mainly interacting with FPR receptor partner family when in the intracellular environment and also as extracellular counterpart [12,15,20,51,52,53] acting in an autocrine and paracrine manner. This contest allowed us to hypothesize that ANXA1 could affect the macrophages by activating its receptor FPR, as described in the hepatocellular carcinoma where this protein is able to enhance the differentiation into M2 macrophages via FPR2 and supports their expression of IL-10 [49].

In consideration of the crucial role of each single-cellular TME component, we studied a three-ways crosstalk among PC cells and TAMs and endothelial cells/fibroblasts. This triple kind of interactions appeared to be notably mediated by ANXA1 which first induces macrophages polarization into M2, and then, this subpopulation in turn supports the other stromal cellular components also by secreting a series of factors. Among them, one important factor is just VEGF-A whose secretion by TAMs interestingly correlates to ANXA1 stimulus [53]. We have also associated this finding to the activation of endothelial cells within the TME, revealed through the induction of migration/invasion processes and mainly the in vitro formation of capillary-like structures. The significant effects of M2 supernatants, obtained in turn by the effects of Ac2-26 and WT EVs, rather than ANXA1 KO ones, suggests the strong action of this protein in the promotion of protumor action by the endothelial cells in TME. 

Additionally, we have found that fibroblasts are importantly influenced by M2 macrophages effects as found through the evaluation of MMP-9 secretion, FAP1α expression, F-actin structures and migration/invasion processes suggesting, once again, and indirect role of ANXA1 [54]. These acquired features are mostly associated to the cancer-associated fibroblasts, (CAFs) which arise in the TME where they promote cancer progression in terms of chemoresistance and by the formation of fibrotic barrier against therapeutic agents [55,56,57,58,59,60,61,62,63,64,65]. 

Furthermore, ex vivo analysis on previously obtained tissue sections, specifically pancreatic tumors and liver metastasis, were warranted to confirm the predominant M2 macrophage infiltration in both cases. In this way, we have confirmed how the presence of ANXA1 can markedly influence the differentiation and/or the recruitment of M2 macrophages into both primary tumor and metastasis.

Thus, this work amplifies the knowledge about the role of ANXA1 as a member of PC EVs by which the protein induces an autocrine and paracrine action. Here, we proved that the paracrine effect is further mediated by the macrophage TME subpopulation. Thus, ANXA1 has been revealed to directly induce M2, as protumor, phenotype and, through these cells, indirectly promotes the activation of PC stroma. 

Taken together, these data make ANXA1 as a key mediator of PC bad behavior suggesting as this protein could become an interesting target to consider in diagnosis/prognosis phases and/or therapy ones.

## 4. Materials and Methods

### 4.1. Cell Culture

MIA PaCa-2 cells (ATCC^®^ CRL-1420; Manassas, VA, USA) were grown as reported in [34]. ANXA1 knockout (KO) MIA PaCa-2 cells were generated as described in [15] and kept in selection by 700 μg/mL neomycin (Euroclone; Milan, Italy). BJ cell line (human immortalized skin fibroblasts, ATCC^®^ CRL-2522^TM^; Manassas, VA, USA) was cultured as in [15]. HUVEC cell line (human umbilical vein endothelial cell) (ATCC^®^ PCS-100-010™; Manassas, VA, USA) was kept in culture until passage 10, as reported in [29]. THP-1 (ATCC^®^ TIB-202; Manassas, VA, USA) cells were maintained in Roswell Park Memorial Institute (RPMI; Euroclone; Milan, Italy) 1640 medium with 1% L-Glutamine, 10% fetal bovine serum (FBS) (Euroclone; Milan, Italy), 1% penicillin and streptomycin and β-mercaptoethanol (0.05 mM; Sigma Aldrich, St. Louis, MO, USA). Cells were grown at 37 °C in air-humidified 5% CO_2_.

### 4.2. Exosomes Isolation

The extracellular vesicles (EVs) enriched in exosomes were obtained from cell culture supernatants as reported in [59]. Previous abundant washing with PBS, the WT and ANXA1 KO MIA PaCa-2 cells (confluence of about 8 × 10^7^ cells in 1.5 × 10^5^ cm^−2^) were cultured for 24 h in DMEM-conditioned medium (without FBS). This medium was collected and processed according to the protocol used in [19,34]. Obtained as reported by Théry and colleagues [59], the supernatant was discarded, and the exosomes pellet was resuspended according to the experimental use.

In total, 20 μg of WT and ANXA1 KO MIA PaCa-2 EVs, previously normalized through the Bradford assay [20,34], were administered to the cells. The normalization was needed to deliver to the cells the same amount of PC EVs, both from WT and ANXA1 KO MIA PaCa-2 cells, in all experiments. Every analysis was performed on fresh isolated fractions.

### 4.3. Macrophages Generation

THP-1 monocytes differentiated into macrophages (M0) via PMA (Sigma Aldrich, St. Louis, MO, USA), 320 nM for 6 h, were allowed to recover for an additional 24 h. One dose of 20 µg of EVs was added for macrophage polarization experiments. After 24 h, the medium was collected, centrifuged to remove cellular component or debris and subjected to the ELISA test for IL10, according to the manufacturer’s guidelines (Elabscience, Houston, TX, USA).

### 4.4. Flow Cytometry

THP-1 monocytes were harvested at a number of 5 × 10^5^/mL, differentiated in macrophages M0, treated with PC EVs and then analyzed for CD80, CD86, CD206 and CD163 expressions as reported in [32]. The pellets were incubated for 1 h at room temperature (RT) in PBS 1x containing FBS (2% *v*/*v*) and antihuman antibody against CD80, CD86, CD206, CD163 (1:100, Santa Cruz Biotechnologies, Dallas, TX, USA) and then for another hour with conjugated secondary antibody (1:100, antimouse). Finally, the cellular markers expression was analyzed by flow cytometer (Becton Dickinson FACScan, Franklin Lakes, NJ, USA) using the Cells Quest program.

### 4.5. Wound-Healing Assay

The confluent monolayer of M0 THP-1, BJ and HUVEC was scraped with a pipette tip to produce a wound. Next, the cells were treated according to the experimental points previously administration of mitomycin C (10 μg/mL, Sigma-Aldrich; Saint Louis, MO, USA) to ensure the block of mitosis. The wounds were photographed and analyzed as reported in [34,60].

### 4.6. Invasion Assay

The invasion capability of cells was performed through the transwell systems (12 mm diameter, 8.0 fim pore size, Corning Incorporated, New York, NY, USA), as previously described [29,30]. The established treatment was added in the lower chambers of each well in experimental points, previously addition of mitomycin C (10 μg/mL, Sigma-Aldrich; Saint Louis, MO, USA) to the arrest of mitosis. Staining and analysis procedures were reported in [18,22].

### 4.7. Clonogenic Assay

The macrophages were seeded 5 × 10^5^ cells/mL in 6-well plates and then were treated with WT and ANXA1 KO EVs and cultured for 8 days in fresh medium. They were subsequently fixed with 4% p-formaldehyde, for 10 min, and then with 100% methanol, for 20 min (both from Sigma-Aldrich; St. Louis, MO, USA). The cellular staining was performed with crystal violet at 0.5% *w*/*v* in a *v*/*v* solution of 20% methanol/distilled water (Merck Chemicals, Darmstadt, Germany) for 30 min at RT. After washing with deionized water, the colonies were photographed, and then, the dye was dissolved in 1% SDS and measured at 570 nm by spectrophotometer [61] (Titertek Multiskan MCC/340; Labsystems, Midland, ON, Canada), as confirmation of the result.

### 4.8. Co-Culture System

**Figure 7:**
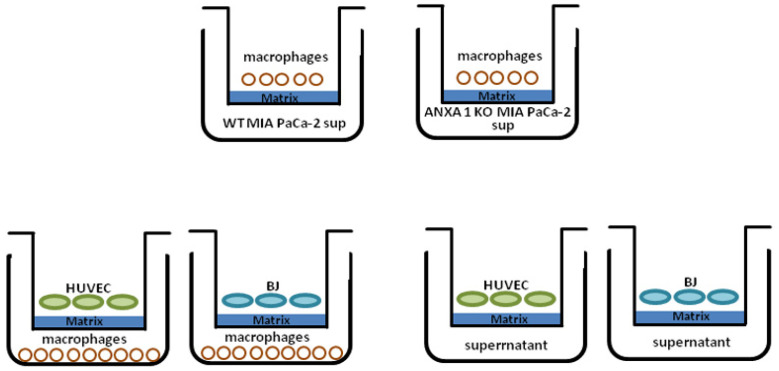
M&M: schematic representation of co-culture system.

Transwell chambers (12 mm diameter, 8.0 fim pore size, Corning Incorporated, New York, NJ, USA) with matrigel coating (BD Biosciences, Franklin Lakes, NJ, USA) were used following the schematic representation reported in Figure 7, to detect macrophage invasion to WT and ANXA1 KO conditioned medium (CM) obtained as the growth medium in contact with WT and ANXA1 KO MIA PaCa-2 cells for 48 h. These CMs were collected and centrifuged at 900× *g* or 10 min to remove the cellular debris. Then, the macrophages were plated in the upper chamber and in the lower chamber the CMs were put. Therefore, this co-culture system was also used to detect the BJ and HUVEC cells invasion to polarized M1 or M2 macrophages, from the upper chamber where cells were seeded at 3 × 10^4^ and 4 × 10^4^/insert, for BJ and HUVEC, respectively. In this case, in the lower chamber, M0 THP-1 pretreated for 24 h with Ac2-26 (1 µM, Tocris Bioscience, Bristol, UK), WT and ANXA1 KO MIA PaCa-2 EVs were plated. After 24 h, all cells were fixed and stained and analyzed as reported in [34].

### 4.9. Confocal Microscopy

HUVEC and BJ cells seeded on glass bottom in multiwell plate were fixed in p-formaldehyde at 4% *v*/*v* in PBS, (Lonza; Basilea, Swiss), permeabilized with Triton X-100 at 0.5% *v*/*v* in PBS (Lonza; Basilea, Swiss) and then blocked with goat serum at 20% *v*/*v* in PBS (Lonza; Basilea, Swiss). Incubation with the respective antibodies against VE-cadherin (mouse monoclonal, 1:100; Santa Cruz Biotechnologies, Dallas, TX, USA), FAP1α (rabbit polyclonal, 1:100; Santa Cruz Biotechnologies, Dallas, TX, USA) were performed for O/N at 4 °C. F-actin detection was evaluated by Phalloidin-FITC (5 μg/mL, Sigma-Aldrich; Saint Louis, MO, USA) for 30 min, at RT in the dark. The staining with conjugated secondary antibodies (1:500, antimouse and antirabbit), the nuclei with DAPI (1:1000) and the subsequent confocal microscope analysis and quantifications were performed as described in [62,63]. In particular, the final images were generated using Adobe Photoshop CS4, version 11.0. Quantifications were performed from multichannel images obtained using a 63× objective using ImageJ, marking either the cell perimeter or the nucleus as the region of interest and calculating integrated densities per area from the appropriate channel. A minimum of 50 cells were analyzed for each data set; the obtained mean value has been used to compare experimental groups.

### 4.10. Gelatin Gel Zymography

The gelatinolytic activity of metalloproteinases was detected by SDS-PAGE zymography as reported in [64]. The serum-free supernatants, mixed with a standard nonreducing loading buffer for SDS-PAGE, were loaded for electrophoretic run, at 125 V, in a gel 10% polyacrylamide and 0.1% gelatin (for protease detection; Sigma Aldrich, St. Louis, MO, USA). After the electrophoresis, the gel was washed in renaturing solution (2.5% Triton X-100) for 1 h to remove SDS, following by incubation in a buffer of digestion (50 mM of Tris-HCl, pH 7.8, 200 mM of NaCl, 5 mM of CaCl_2_ and 5 mM of ZnCl_2_) for degradation of the substrate for 18 h, at 37 °C. The gels staining was with Coomassie Brilliant Blue R-250 (Sigma Aldrich, St. Louis, MO, USA) and then washed with destaining solution (10% methanol and 5% acetic acid) to reveal the areas of digestion such as a light band.

### 4.11. ELISA for VEGF-A

After treatments with WT and ANXA1 KO EVs, THP-1 supernatants were collected, and the secreted VEGF-A amount was detected through the human VEGF-A ELISA kit, following the manufacturer’s instructions (Invitrogen, Carlsbad, CA, USA).

### 4.12. Tube Formation Assay

The in vitro angiogenesis has been performed as reported in [65]. After 12 h, the 10× images were acquired by EVOS^®^ optical microscope (Life Technologies Corporation, Carlsbad, CA, USA) and analyzed both for length and the number of branches by the ImageJ software (NIH, Bethesda, MD, USA) (Angiogenesis Analyzer tool for ImageJ).

### 4.13. Western Blotting

Proteins extracted from cells were examined by SDS-PAGE. Protein content was estimated according to Biorad protein assay (BIO-RAD, Hercules, CA, USA), as previously described [34]. We have analyzed primary antibodies against FAP1α (rabbit polyclonal (1:500; Santa Cruz Biotechnologies, Dallas, TX, USA) and tubulin (mouse monoclonal 1:1000; Sigma-Aldrich; St. Louis, MO, USA). The blots were exposed to Las4000 (GE Healthcare Life Sciences; Little Chalfont, UK).

### 4.14. H&E Tissue Staining and Tissue Immunofluorescence

Frozen samples of tumor and metastasis tissues were obtained from our previous in vivo study on mice [15]. In brief, SCID mice (6–8 week-old females) were obtained from Charles River (Italy) and bred under pathogen-free conditions in the Animal Facility of the University of Salerno. The orthotopic implantations were performed in the pancreas by using WT and ANXA1 KO MIA PaCa-2 cells. After 5 weeks from the implantation, mice were sacrificed, and organs (pancreas and livers) were excised, weighed and analyzed. Metastases lesions on the liver surface were observed and quantified by gross anatomy using a dissecting microscope. The slice sections of 6 μm, laying on superfrost slides (Thermo Scientific, Thermo Fisher Scientific; Waltham, MA, USA), were processed for hematoxylin and eosin (H&E) staining, as reported in [13]. The images were taken through the Axio Observer microscope (20×) (Carl Zeiss MicroImaging GmbH; Jena, Germany) and analyzed using ImageJ software (NIH, Bethesda, MD, USA).

Next, the sections were fixed in a solution of p-formaldehyde (4% *v*/*v* in PBS; Lonza), were permeabilized or not with Triton X-100 (0.5% *v*/*v* in PBS; Lonza), blocked with fetal bovine serum (FBS) (20% *v*/*v* in PBS, 0.5% Triton X-100; Lonza, Basilea, Swiss) and then incubated with anti-CD80 (mouse monoclonal, 1:100; Santa Cruz Biotechnologies, Dallas, TX, USA), anti-CD86 (mouse monoclonal, 1:100; Santa Cruz Biotechnologies, Dallas, TX, USA), anti-CD163 (mouse monoclonal, 1:100; Santa Cruz Biotechnologies, Dallas, TX, USA), anti-CD206 (mouse monoclonal, 1:100; Santa Cruz Biotechnologies, Dallas, TX, USA) in determined solution 0.2% Triton X-100, 3% FBS, 0.02% NaN3, overnight at 4 °C. Then, they were incubated with antimouse AlexaFluor (488 and/or 555; 1:500; Molecular Probes; Waltham, MA, USA) for 2 h at RT. After two washing steps, the slice sections were mounted, and the images were acquired using a Leica SP8 confocal microscope (Leica Microsystems, Wetzlar, Germany). The densitometry analysis was performed as reported above; in this case, a 40× objective was used, and the whole slide was evaluated to count nuclei and the related protein signal for each data set. The obtained mean value was used to compare experimental groups. Fifteen sections from six animals (three for each condition of implantation) were selected.

### 4.15. Statistical Analysis

All the data and statistical analysis were made with Microsoft Excel. We reported the number of independent repetitions and p values in the legends of the figures for each experiment. All results are the mean ± standard deviation of at least three experiments performed in triplicate. The statistical data analysis were performed thanks to the two-tailed t test comparing two variables, and the differences were considered significant if *p* < 0.05, *p* < 0.01 and *p* < 0.001.

## 5. Conclusions

In recent years, it has become clear the fundamental role of TME in tumor progression and metastasis. In this setting, one of the important of cell-to-cell communication is represented by extracellular vesicles by which cancer cells can activate stroma elements. In parallel, our previous works demonstrated that ANXA1 promotes PC progression via EVs inducing a mesenchymal phenotype on fibroblasts and endothelial cells. In this study, we have shown that ANXA1/EVs complex regulates macrophage polarization into the M2 subpopulation, by which they could promote metastasis. Thus, the complex ANXA1/EVs is involved in modulating the interaction between TAMs and CAFs, that regulate tumor-associated inflammation and between TAMs and endothelial cells that improve the angiogenesis. Moreover, since M2 cells are also involved in drug resistance [4], targeting the interaction between ANXA1/EVs and macrophages might be a potential therapeutic strategy for PC. Further investigations are necessary to confirm these issues mainly focused on EVs deriving from PC on other cell populations surrounding the primary tumor. Particularly, it would be interesting to perform in vivo studies to explore the effects of this complex by directly implanting WT and ANXA1 KO EVs in mice pancreas. These future analyses could also contribute to specifying the mechanism by which these effects are triggered, mainly focusing on FPRs evaluation as ANXA1 peculiar receptor partners.

## Data Availability

The data presented in this study are available upon request from the corresponding author.
